# Relationship between Parenting Style and Risk of Attention Deficit Hyperactivity Disorder in Elementary School Children

**DOI:** 10.21315/mjms2022.29.4.14

**Published:** 2022-08-29

**Authors:** Alma Rossabela Setyanisa, Yunias Setiawati, Irwanto Irwanto, Izzatul Fithriyah, Satria Arief Prabowo

**Affiliations:** 1 Medical Program, Faculty of Medicine, Universitas Airlangga, Surabaya, Indonesia; 2 Department of Psychiatry, Faculty of Medicine, Universitas Airlangga/Dr. Soetomo Academic Medical Center Hospital, Surabaya, Indonesia; 3 Department of Child Health, Faculty of Medicine, Universitas Airlangga/Dr. Soetomo Academic Medical Center Hospital, Surabaya, Indonesia; 4 Vaccine Centre and Tuberculosis Centre, Faculty of Infectious and Tropical Disease, London School of Hygiene and Tropical Medicine, University of London, London, United Kingdom

**Keywords:** attention deficit hyperactivity disorder, parenting style, elementary school, children, child well-being

## Abstract

**Background:**

Attention deficit hyperactivity disorder (ADHD) is a neurodevelopmental disorder in a child with three symptoms, which include inattention, hyperactivity and impulsiveness that may persist into adulthood for some conditions. Parenting style is thought to be one part that determines the risk of ADHD in children. This study aims to analyse the relationship between parenting styles and the risk of ADHD in children.

**Methods:**

Employing a cross-sectional design, this study was conducted in Surabaya from November 2020 to January 2021. The respondents of the study were parents who had children at risk of ADHD with a total of 55 samples recruited using the purposive sampling technique. The questionnaires used are the demographic questionnaire, Abbreviated Conners Rating Scale (ACRS), and the Parenting Style Questionnaire for Children and Adolescents (KPAA), which were filled out online by the respondents. The data were processed and analysed using the bivariate analysis test, Pearson’s chi-squared test, which has a significant value if the *P*-value < 0.05.

**Results:**

The majority of the parents used the authoritative style (94.5%). There was a significant relationship between parenting style and the risk of ADHD in children with *P* < 0.001 for authoritarian and permissive styles and *P* = 0.005 for an authoritative style.

**Conclusion:**

There is a significant relationship between parenting style and the risk of ADHD in children. This indicates the importance of early diagnosis of ADHD and treating the children with ADHD in the context of family environment, especially from parenting style.

## Introduction

Attention deficit hyperactivity disorder (ADHD) is a neurodevelopmental disorder causing emotional and behavioural disorders in children. The three symptoms of ADHD are inattention, hyperactivity and impulsivity that typically occur before a child turns 12 years old and in some conditions can persist into adulthood. The symptoms of ADHD occur in two different situations and these conditions can cause problems in social, educational and work aspects. In the United States, the prevalence of ADHD ranges from 3% to 7% (1).

One of the risk factors that influence ADHD in children is the parenting style of parents who are playing their role poorly and the lack of positive parenting practices (2). Parents who have children with ADHD tend to practice a negative parenting approach, have more control towards child behaviour and give more punishment because of their child’s externalising behaviour. This condition will aggravate ADHD in children and will lead to other secondary behaviours such as rebelliousness and aggressiveness (3). The current coronavirus disease 2019 (COVID-19) pandemic has made children have to study at home through online learning. This situation will make children feel bored and the condition of their parents will be affected because they have to make adjustments to the mentoring during the learning process at home by supervising learning activities as well as being a teacher during learning at home (4–5). In Indonesia, not many studies focus on the relationship between parenting style and ADHD in children, even though ADHD in children can harm them and those around them. This study aims to determine the relationship between parenting styles in children and the risk of ADHD to get more information about parenting in children with ADHD so that the risk of ADHD in children can be minimalised.

## Methods

### Data Collection

This study involved students aged between 9 years old and 12 years old from five schools in Surabaya, namely: i) Semolowaru I/261 Elementary School; ii) Semolowaru IV/614 Elementary School; iii) Keputih 245 Elementary School; iv) Kertajaya Elementary School and v) Baratajaya Elementary School. Research data were obtained from the Pediatric Psychiatry Unit of Dr. Soetomo General Academic Hospital, Surabaya, which reported 72 cases of ADHD in 2008 and 103 cases in 2009 (6). Based on the data, this research was conducted in Surabaya, with ADHD cases in children increasing every year. The population included children aged less than 12 years old referring to ADHD conditions that typically occur in the age group before 12 years old (1). The total population of this study consisted of 449 parents of students from the 4th until 6th grade recruited using purposive sampling technique. The inclusion and exclusion criteria were determined and resulted in the sample of 55 parents of students who were suitable with the calculation minimum of sample size (Equation 1).

The study design was cross-sectional and all data were taken using a questionnaire that the parents of the students filled in through an online platform from November 2020 to January 2021.


Equation 1 $$n\geq {\left(\frac{Z_{1-\frac{\alpha }{2}}+Z_{1-\beta }}{\frac{1}{2}{\text{log}}_{e}\frac{1+r}{1-r}}\right)}^{2}+3$$(
7)

Calculation of the minimum sample used the following parameters:


α=0.05,β=0.2,r=0.5

The demographic questionnaire contains data of students and data of parents. The Abbreviated Conners Rating Scale (ACRS) questionnaire which consists of 10 questions was used to assess the risk of ADHD in children listed in the Stimulation, Detection, and Early Intervention of Child Growth and Development (SDIDTK) book (8). The validity value of the ACRS questionnaire is 93.94% and the reliability is 90.91% (9). Children are considered to have a high risk of ADHD if the total score obtained is more than or equal to 13 (8). Parenting Style Questionnaire for Children and Adolescents (KPAA) was employed to identify the types of parenting that parents predominantly apply to their children; there are four types of parenting: i) authoritative; ii) authoritarian; iii) permissive and iv) mixed (10). This questionnaire consists of 26 questions. This questionnaire has a validity value of 0.8367 and Cronbach’s alpha value produces a reliability of 0.8342 (10).

### Statistical Analysis

The data were processed and analysed using univariate analysis techniques to see the frequency and percentage of variables and bivariate analysis to see the relationship between parenting style and risk of ADHD in children using the Pearson’s chi-squared test, considered significant if the *P*-value was < 0.05.

## Results

The socio-demographic data of children and parents showed that male children dominated, which was 56.4% and the average age was of 10 years old, which was 32.7%. Most parents were between 31 years old and 40 years old (60%). Most dominant education background is senior high school (70.9%). Most of the respondents were housewives (47.3%) and the majority income is between IDR0 and IDR1,500,000 (50.9%) (Table 1). The risk assessment for ADHD was done using the ACRS questionnaire with a cut-off score of 13. The prevalence of ADHD risk in children in this study resulted in 55 children (12.2%) from a total of 449 children who exceeded the specified cut-off value, indicating that they were classified as having a high risk for ADHD (Table 2).

There were two types of parenting style among parents with children at risk for ADHD identified in this study, which were authoritative and authoritarian parenting styles. The parenting style with the highest frequency was authoritative (94.5%) (Table 3). An analysis on the relationship between parenting style and risk of ADHD in the children revealed a significant relationship between each type of parenting style and the risk of ADHD in children (*P* = 0.000 for authoritarian and permissive style, *P* = 0.005 for authoritative style) (Table 4).

## Discussion

### Children with Risk of ADHD

The results of this study found 55 samples of children who were at high risk of ADHD in this study (Table 2) with a greater percentage of males than females (56.4%; 43.6%) (Table 1). Following research results in Bali and Surabaya, the frequency of sex in ADHD children was more male than the female, with a ratio of 2:1 (11–12). The theory also states that ADHD will be more common in boys because boys are more likely to be more challenging and aggressive (12).

The age selection of children in this study follows the Diagnostic and Statistical Manual of Mental Disorders 5 (DSM-5) which explains that ADHD symptoms will be seen before turning 12 years old of age (1). The results were obtained where the age of 10 years old had the most frequency and was followed by 11 years old, 12 years old and 9 years old (Table 1). Novriana (13) in her research also found that children with ADHD were more common in the 11 years old–13 years old of age range. The risk of ADHD increases with older children. In several situations, ADHD can persist into adulthood, especially when the ADHD is not detected as early as possible and is not treated correctly. In that case, it will cause other severe conditions, such as education and social relationship problems, depression, substance abuse, violation, accidental injury and failure to do a job (1, 14). This also shows the importance of early detection in children so that ADHD conditions can be reduced with earlier management.

### Overview of Parenting Styles

The results of this study indicate that the types of parenting styles applied in this study are authoritative and authoritarian parenting with a greater frequency of authoritative parenting than authoritarian parenting, which is 94.5% (Table 3). Following previous research in Manado, authoritative parenting was more frequently applied to children with ADHD, followed by permissive and authoritarian parenting (15). Authoritative parenting is characterised by the attitude of parents who have discussions more frequently with their children to respond to their children’s behaviours (16).

### Parenting Style and Risk of ADHD

The bivariate analysis test between parenting styles and the risk of ADHD in children with the Pearson’s chi-squared test technique obtained *P*-value = 0.000 and *P*-value = 0.005 (*P* < 0.05). It can be concluded that there is a significant relationship between parenting styles and ADHD in children (Table 4). In the research conducted by Rusnoto (17) it was found that there was no significant relationship between parenting styles for children with ADHD. This can occur because the research uses different instruments, research locations and populations, which affect the sample’s characteristics. In addition, the factors that influence ADHD in children are still not known. However, the most dominant theory states that genetic factors related to the neurotransmitters dopamine and norepinephrine influence the onset of ADHD in children (18). Parenting style is a risk factor that influences the emergence of ADHD in children, which can worsen the condition and lead to other secondary behavioral problems (2). So, different results could be obtained because many factors influence the incidence of ADHD in children and further research could help determine what factors influence the condition of ADHD in children.

The three symptoms of ADHD ranging from inattention are indicated by decreased attention when in situations for a long time, both when the child is in learning and playing conditions. Impulsiveness is shown by the characteristics of a child who often imposes his own will and likes to interrupt others. Meanwhile, hyperactivity can be in the form of behaviour of children who cannot maintain their position in certain situations for a long time and the conditions of the surrounding environment. If not treated properly, these three symptoms can persist until adulthood and will cause harm to both the individual and the surrounding environment (1). The risk of ADHD in children is influenced by risk factors such as genetic and environmental interactions (19). One of the main topics of discussion in this study is the types of parenting that parents use while taking care of their children.

### Authoritarian Style and Risk of ADHD

There is a relationship between authoritarian parenting and the risk of ADHD in children (Table 4). This result is in line with a previous study in Egypt that there is a significant relationship between ‘negative’ parenting or authoritarian parenting and ADHD severity (20). Authoritarian parenting is more widely applied to children at risk for ADHD than to parents of children who are not at risk for ADHD. Authoritarian parenting is a type of parenting that tends to force children and show a firm attitude towards children and require children to obey the words of their parents (16). The parenting style of parents who are doing their role poorly and the lack of positive parenting practices will be a risk factor for the onset of ADHD in children (2). Parents who often give threats or punishments to their children will be a different risk factor for the emergence of the aggressive nature of the child (21). Authoritarian parenting is a type of parenting that should not be applied by parents where the impact of the child being less attention from parents in parenting will reduce cognitive stimulation in children (22). This will also affect stress conditions in children which will affect the neurotransmitters dopamine and norepinephrine causing symptoms of hyperactivity in children (18). This will increase the risk of ADHD.

### Authoritative Style and Risk of ADHD

Authoritative parenting prioritises collaboration and communication to solve problems between children and parents (16). However, the results of this study revealed a relationship between authoritative parenting and the risk of ADHD in children. This can be a particular concern because even though authoritative parenting is a parenting style that should applied by parents, children still face the risk of experiencing ADHD. Researchers predict that this may happen because of the urgency of the COVID-19 pandemic that was happening when the research is being conducted. Parents as caregivers must make adjustments to take care of their children, especially when accompanying and helping children learn at home. The pandemic causes children to become bored more easily and aggressive to behavior in daily activities (5). Parents experiencing confusion in the parenting styles that will be given to their children and difficulties in teaching and adjusting to their children will result in negative or inappropriate parenting and lead to a higher risk of ADHD (12, 23).

### Permissive Style and Risk of ADHD

There is a significant relationship between permissive parenting and the risk of ADHD in children. This result is supported with a study conducted by Moghaddam et al. (24) revealing that in the ADHD group, it was found that the number of permissive parenting patterns was higher after authoritative parenting. Permissive parenting fulfills all children’s wants and grants freely. Children who are cared for with permissive parenting will become individuals who always impose their will, tend to underestimate small things, grow more impulsive and are less responsible (25). Children’s behaviour will be easier to control if parents provide good support to their children during parenting (26). Meanwhile, parents who apply permissive parenting typically do not understand the conditions experienced by their children, and the relationship between parents and children is also not as good as that of parents applying other types of parenting because parents do not pay attention to or reject the symptoms of ADHD in children (25). Therefore, the condition of ADHD becomes uncontrollable, and there are still many undetected risks of ADHD because parents think their child is not experiencing something serious even though ADHD conditions in children require a good relationship between parents and children and can understand the condition of ADHD.

However, the result of this study can be optimised by considering the following limitations: (i) using a larger sample size so that it can produce more general conclusions not only on the population or sample used; (ii) observation on the socio-demographic conditions of parents on the risk of ADHD in children. This is not discussed further in this study considering the wide range of inclusion criteria used.

## Conclusion

There is a significant relationship between parenting styles and ADHD in children. The dominant parenting style in this study is authoritative parenting. Parents who have children at high risk of ADHD mostly applied authoritarian parenting. Lack of parental attention through parenting can increase dopamine and norepinephrine levels in children, which is one of the causes of hyperactivity and will increase the risk of ADHD in children. More information about the condition of ADHD in children, the types of parenting as well as the advantages and disadvantages of each type of parenting is needed, so that parents can apply parenting styles appropriate to the child’s condition. Future research should focus on risk factors other than parenting that are thought to affect the risk of ADHD in children so that this condition can be minimised by early management.

## Figures and Tables

**Table 1:** Characteristics socio-demographic of children and parents

Children	Category	Frequency *n* (%) (*N* = 55)
Gender	Boys	31 (56.4)
	Girls	24 (43.6)
Age (years old)	9	4 (7.3)
	10	18 (32.7)
	11	17 (30.9)
	12	16 (29.1)
Education	Junior high school	7 (12.7)
	Senior high school	39 (70.9)
	Associate degree	1 (1.8)
	Bachelor degree	8 (14.5)
Age (years old)	21–30	3 (5.5)
	31–40	33 (60)
	41–50	17 (30.9)
	51–60	2 (3.6)
Occupation	Housewife	26 (47.3)
	Entrepreneur	10 (18.2)
	Private	7 (12.7)
	Labourer	4 (7.2)
	Government employees	3 (5.5)
	Others	5 (9)
Income (Rupiah)	0–1,500,000	28 (50.9)
	1,500,001–2,500,000	12 (21.8)
	2,500,001–3,500,000	8 (14.5)
	More than 3,500,000	7 (12.7)

**Table 2:** Prevalence the risk of ADHD in children

Category	Frequency *n* (%) (*N* = 449)
≥ 13	55 (12.2)
< 13	394 (87.8)

**Table 3:** Overview of parenting style and risk of ADHD in children

	Category	Frequency *n* (%) (*N* = 55)
Parenting style	Authoritative	52 (94.5)
	Authoritarian	3 (5.5)

**Table 4:** Relationship between parenting style and risk of ADHD in children

		Parenting style						*n*

		Authoritative *n* (%)	*P*-value	Authoritarian *n (%)*	*P*-value	Permissive *n* (%)	*P*-value	
Risk of ADHD	≥ 13	52 (94.5)	0.005*	3 (5.5)	0.000*	0 (0)	0.000*	55
	< 13	391 (99.2)		1 (0.3)		2 (0.5)		394

Notes:

* Pearson’s chi-squared test; significant level at *P* < 0.05

## References

[1] American Psychiatric Association (2013). Diagnostic and statistical manual of mental disorders.

[2] Teixeira MC, Marino RL, Carreiro LR (2015). Associations between inadequate parenting practices and behavioral problems in children and adolescents with attention deficit hyperactivity disorder. Sci World J.

[3] Setiawati Y, Mukono HJ, Wahyuhadi J, Warsiki E (2018). The relationship between severity of Attention Deficit Hyperactivity Disorder (ADHD) with maternal anxiety. Health Notion.

[4] Surabaya Walikota (2020). Peraturan Walikota Surabaya Nomor 16 Tahun 2020 tentang pedoman pembatasan sosial berskala besar dalam penanganan Coronavirus disease 2019 (COVID-19) di Kota Surabaya [Internet];.

[5] Zhou X, Snoswell CL, Harding LE, Bambling M, Edirippulige S, Bai X (2020). The role of telehealth in reducing the mental health burden from COVID-19. Telemed e-Health.

[6] Saputro D (2009). ADHD (Attention Deficit Hyperactivity Disorder).

[7] Sternberg RJ, Sternberg K (2017). Cognitive psychology.

[8] Kementrian Kesehatan RI (2016). Pedoman pelaksanaan stimulasi, deteksi dan intervensi dini tumbuh kembang anak ditingkat pelayanan kesehatan dasar. Kementrian Kesehatan RI.

[9] Sasanti Y (1998). Penentuan validitas dan reliabilitas Abbreviated Conners Teacher Rating Scale (ACTRS) sebagai penyaring kegiatan hiperaktivitas. Tesis.

[10] Ismail RI (2015). Kuisioner pola asuh anak dan remaja.

[11] Esalini IAP, Lesmana CB (2019). Tingkat kemandirian anak Attention Deficit Hyperactivity Disorder dengan terapi perilaku di Yayasan Mentari Fajar Jimbaran Badung. E-Jurnal Medika Udayana.

[12] Sasono CK, Setiawati Y, Irwanto (2019). Correlation between children’s temperament and risk factor of Attention Deficit/Hyperactivity Disorder in elementary school. Biomol Health Sci J.

[13] Novriana DE, Yanis A, Masri M (2014). Prevalensi gangguan pemusatan perhatian dan hiperaktivitas pada siswa dan siswi Sekolah Dasar Negeri Kecamatan Padang Timur Kota Padang Tahun 2013. Jurnal Kesehatan Andalas.

[14] Children and Adults with Attention-Deficit/Hyperactivity Disorder (CHADD) (2021). About ADHD [Internet].

[15] Kaunang NE, Munayang H, Kaunang TM (2016). Pola asuh pada anak gangguan pemusatan perhatian dan hiperaktivitas di Kota Manado. e-Clinic (eCl).

[16] Baumrind D (1966). Effects of authoritative parental control on child behavior. Child Dev.

[17] Rusnoto (2016). Hubungan pola asuh dan riwayat merokok dengan resiko Attention Deficit Hyperactivity Disorder (ADHD) pada anak pra sekolah di Tk Kasian. Jurnal Ilmu Keperawatan dan Kebidanan.

[18] Dark C, Homman-Ludiye J, Bryson-Richardson RJ (2018). The role of ADHD associated genes in neurodevelopment. Dev Biol.

[19] NHFA, Setiawati Y (2017). Interaksi faktor genetik dan lingkungan pada Attention Deficit/Hyperactivity Disorder (ADHD). Jurnal Psikiatri Surabaya.

[20] Elemam M, Abdelmaksoud A, Abo-Elabbas M (2020). Effect of parenting style on severity of Attention-Deficit/Hyperactivity Disorder among children attending Al-Azhar University Hospital, New Damietta. Int J Med Arts.

[21] Setiawati Y, Mirantri K, Mukono HJ, Wahyuhadu J, Warsiki E (2020). Correlation between parenting styles and peer attachment with aggressive behavior potentials in adolescent boys. Indian J Forensic Med Toxicol.

[22] Dodangi N, Vameghi R, Habibi N (2017). Evaluation of knowledge and attitude of parents of attention deficit/hyperactivity disorder children towards Attention Deficit/Hyperactivity Disorder in clinical samples. Iranian J Psychiatry.

[23] Karki U, Dhonju G, Kunwar Ar (2020). Parenting during the COVID-19 pandemic. J Nepal Med Assoc.

[24] Moghaddam FM, Assareh M, Heidaripoor A, Eslami Rad R, Pishjoo M (2013). The study of comprising parenting style between children with ADHD and normal children. Arch Psychiatry Psychother.

[25] Setiawati Y, Mirantri K, Syulthoni ZB (2020). The relationship between mother’s parenting patterns and aggressive behavior of adolescent son in risk environments. Eurasia J Biosci.

[26] Brown SM, Doom JR, Lechuga-Peña S, Watamura SE, Koppels T (2020). Stress and parenting during the global COVID-19 pandemic. Child Abuse Neglect.

